# The two *Rasamsonia emersonii* α-glucuronidases, *Re*GH67 and *Re*GH115, show a different mode-of-action towards glucuronoxylan and glucuronoxylo-oligosaccharides

**DOI:** 10.1186/s13068-016-0519-9

**Published:** 2016-05-18

**Authors:** Patricia Murciano Martínez, Maaike M. Appeldoorn, Harry Gruppen, Mirjam A. Kabel

**Affiliations:** Laboratory of Food Chemistry, Wageningen University, Bornse Weilanden 9, 6708 WG Wageningen, The Netherlands; DSM Biotechnology Center, Alexander Fleminglaan 1, 2613 AX Delft, The Netherlands

**Keywords:** Biorefinery, α-Glucuronidase, GH67, GH115, *Rasamsonia emersonii*, Xylo-oligosaccharides

## Abstract

**Background:**

The production of biofuels and biochemicals from grass-type plant biomass requires a complete utilisation of the plant cellulose and hemicellulosic xylan via enzymatic degradation to their constituent monosaccharides. Generally, physical and/or thermochemical pretreatments are performed to enable access for the subsequent added carbohydrate-degrading enzymes. Nevertheless, partly substituted xylan structures remain after pretreatment, in particular the ones substituted with (4-*O*-methyl-)glucuronic acids (UA_me_). Hence, α-glucuronidases play an important role in the degradation of UA_me_xylan structures facilitating the complete utilisation of plant biomass. The characterisation of α-glucuronidases is a necessity to find the right enzymes to improve degradation of recalcitrant UA_me_xylan structures.

**Results:**

The mode-of-action of two α-glucuronidases was demonstrated, both obtained from the fungus *Rasamsonia emersonii*; one belonging to the glycoside hydrolase (GH) family 67 (*Re*GH67) and the other to GH115 (*Re*GH115). Both enzymes functioned optimal at around pH 4 and 70 °C. *Re*GH67 was able to release UA_me_ from UA_me_-substituted xylo-oligosaccharides (UA_me_XOS), but only the UA_me_ linked to the non-reducing end xylosyl residue was cleaved. In particular, in a mixture of oligosaccharides, UA_me_XOS having a degree of polymerisation (DP) of two were hydrolysed to a further extent than longer UA_me_XOS (DP 3–4). On the contrary, *Re*GH115 was able to release UA_me_ from both polymeric UA_me_xylan and UA_me_XOS. *Re*GH115 cleaved UA_me_ from both internal and non-reducing end xylosyl residues, with the exception of UA_me_ attached to the non-reducing end of a xylotriose oligosaccharide.

**Conclusion:**

In this research, and for the first time, we define the mode-of-action of two α-glucuronidases from two different GH families both from the ascomycete *R. emersonii*. To date, only four α-glucuronidases classified in GH115 are characterised. *Re*GH67 showed limited substrate specificity towards only UA_me_XOS, cleaving UA_me_ only when attached to the non-reducing end xylosyl residue. *Re*GH115 was much less substrate specific compared to *Re*GH67, because UA_me_ was released from both polymeric UA_me_xylan and UA_me_XOS, from both internal and non-reducing end xylosyl residues. The characterisation of the mode-of-action of these two α-glucuronidases helps understand how *R. emersonii* attacks UA_me_xylan in plant biomass and the knowledge presented is valuable to improve enzyme cocktails for biorefinery applications.

**Electronic supplementary material:**

The online version of this article (doi:10.1186/s13068-016-0519-9) contains supplementary material, which is available to authorized users.

## Background

For the production of biofuels and chemicals from plant biomass, a complete utilisation of the cellulose and hemicellulose present is desired. The degradation of these polymers is commonly approached via a physical and/or thermo-assisted chemical pretreatment, followed by enzymatic hydrolysis. In grass-type feedstocks, glucuronoarabinoxylan (GAX) is the major hemicellulose. It is constituted of a β-(1 → 4) linked xylopyranosyl backbone, substituted by side groups, such as *O*-acetyl groups, arabinofuranosyl residues and 4-*O*-methyl-α-d-glucopyranosyl uronic acids. The occurrence of substituents in the xylan backbone is highly dependent on the feedstock used. In addition, the abundance and distribution of substituents can be affected by the type and severity of the pretreatment performed [1]. For example, *O*-acetyl groups and arabinosyl residues are released during hydrothermal pretreatments catalysed by alkali or acids [2–4]. However, glucuronic acid (UA) and its 4-*O*-methyl etherified derivative (UA_me_) are hardly removed from the xylan backbone during such treatments [1, 5]. Therefore, in commercial enzyme cocktails α-glucuronidases are crucial in addition to endo-xylanases and β-xylosidases, to achieve a complete hydrolysis of monosaccharides.

Such commercial enzyme cocktails mostly contain enzymes produced by ascomycetes, like *Aspergillus* species and *Trichoderma* species. In addition, the ascomycete *Rasamsonia emersonii* is a candidate for the production of (hemi-) cellulolytic enzymes. In this research, α-glucuronidases from *R. emersonii* were studied.

In fungi, α-glucuronidases are classified based on the Carbohydrate-Active enZymes database [6, 7] in two glycoside hydrolase (GH) families, which are GH67 and GH115. In the genome of 38 different basidiomycetes, only genes encoding GH115 are described and zero encoding GH67, which indicates that GH115 is preferred over to GH67 in basidiomycetes [8]. For ascomycetes, like for various *Aspergillus* strains, such a distinct choice between GH115 and GH67 is not present. Depending on the strain, genes are present encoding only GH67 or both GH67 and GH115 [8]. In addition to such genome annotations, mainly based on putative functions, it is even more valuable to characterise the mode-of-action of the enzyme proteins corresponding to the annotated genes. The mode-of-action of GH67 α-glucuronidases is well known, because many GH67 α-glucuronidases have been characterised and all are able to release UA_me_ linked to the non-reducing xylosyl end in xylo-oligosaccharides (UA_me_XOS) [9–11]. GH67 α-glucuronidases are not able to release UA_me_ from polymeric glucuronoxylan (UA_me_xylan) [10, 11]. In contrast with the GH67 α-glucuronidases, the mode-of-action of only a limited number of GH115 α-glucuronidases has been described. Only four α-glucuronidases have been biochemically characterised so far, one isolated from the basidiomycete *Schizophyllum commune,* one from the ascomycete *Pichia stipitis* and two from the bacteria *Streptomyces pristinaespiralis and Bacteroides ovatus*. All four are able to release UA_me_ from UA_me_xylan [12–15]. In addition, all four GH115 α-glucuronidases are able to release UA_me_ from UA_me_XOS, linked to either internal or to the non-reducing end xylosyl residues [14, 16].

In this research, for the first time, two purified α-glucuronidases, both from the ascomycete *R. emersonii*, belonging to either GH67 or GH115, are extensively characterised for their mode-of-action towards UA_me_xylan and UA_me_XOS. *Rasamsonia emersonii* grows well at temperatures around 45–50 °C and not able to grow at 25 °C. *Rasamsonia emersonii* is, therefore, catalogued as thermophilic [17]. It is hypothesised, based on the above-described literature findings on the mode-of-action of α -glucuronidases, that the two enzymes studied show a different mode-of-action towards the substrates studied at 65 °C and pH 4.5.

## Results and discussion

**Fig. 1:**
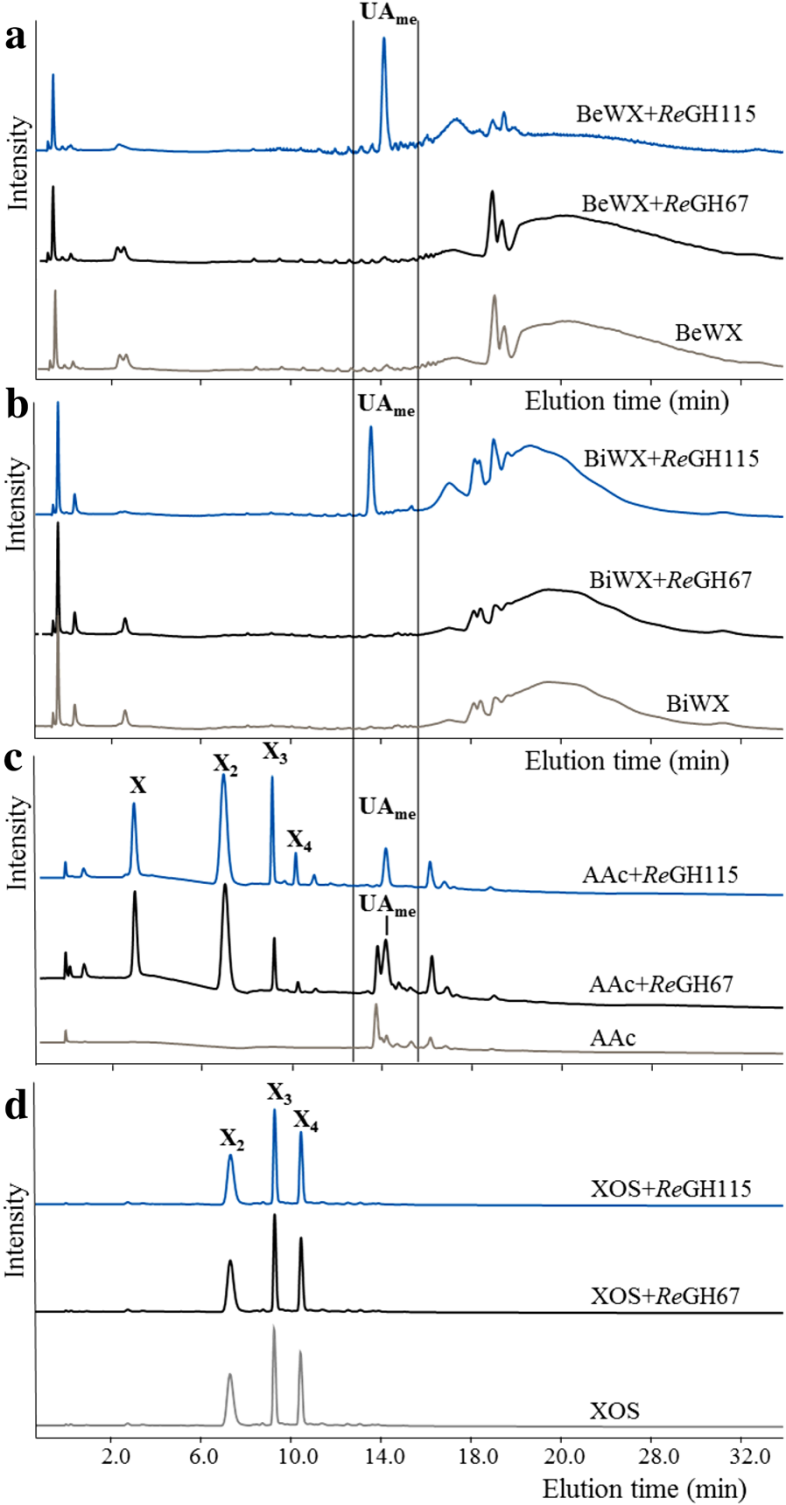
High-performance anion exchange chromatograms of beechwood xylan (BeWX) (**a**), birchwood xylan (BiWX) (**b**), the aldouronic acid mixture (AAc) (**c**), and xylo-oligosaccharides (XOS) (**d**) before and after incubation with *Re*GH115 and *Re*GH67. *X* xylose, *X*_*2*_ xylobiose, *X*_*3*_ xylotriose, *X*_*4*_ xylotetraose, *UA*_*me*_ 4-*O*-methylglucuronic acid

Purification of the α-glucuronidases ReGH67 and ReGH115. The crude enzyme extracts obtained from *Aspergillus niger*, having overexpressed ReGH67 or ReGH115, were purified by a 2-step size exclusion chromatography; a subsequent cation exchange step was applied to the SEC 2-fraction of ReGH115 (data not shown). SDS-PAGE of the purified ReGH67 and ReGH115 showed bands of a molecular mass of 100 and 150 kDa, for ReGH67 and ReGH115, respectively, compared to the marker (data not further shown). These masses differed with the predicted masses of the enzymes based on their amino acid sequences, which were 91 and 111 kDa for ReGH67 and ReGH115, respectively. Possibly, ReGH67 and ReGH115 were glycosylated, which could have resulted in the higher molecular mass analysed. In addition, it was shown that no xylanase activity was present in the purified ReGH67 and ReGH115; neither towards 4-*O*-methylglucuronoxylan (Fig. 1), nor towards linear xylo-oligosaccharides (XOS) (Additional file 1: Figure S1).

### Activity of *Re*GH115 and *Re*GH67 towards 4-*O*-methylglucuronoxylan

To date, only four α-glucuronidases belonging to GH115 have been characterised, of which only one originates from an ascomycete, while many GH67 α-glucuronidases have been studied. Therefore, we are in particular interested in the mode-of-action of *Re*GH115, although *Re*GH67 is also of interest as this enzyme originates from the same ascomycete *R. emersonii* as the studied *Re*GH115.

**Fig. 2:**
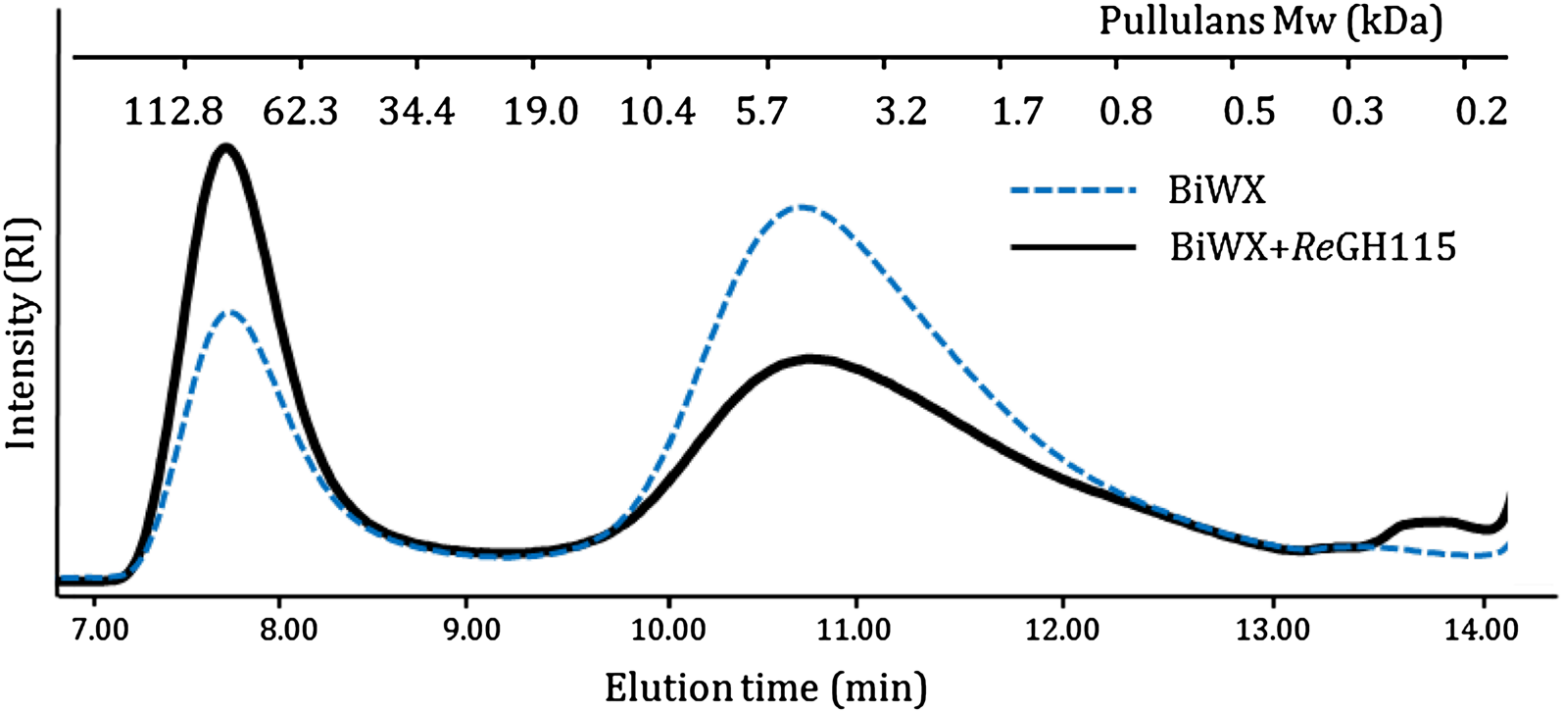
High-performance size exclusion chromatograms of birchwood xylan before (*solid line* in *black*) and after (*dotted line* in *blue*) hydrolysis with *Re*GH115

The activity of the two purified α-glucuronidases was first tested towards BeWX and birchwood xylan (BiWX), which are polymeric xylans constituted of β-(1 → 4) linked xylosyl residues substituted with α-(1 → 2) linked 4-*O*-methylglucuronic acids (UA_me_). The UA_me_ substitution pattern of BiWX has been reported to be more blockwise compared to BeWX, which is more random [18]. BiWX and BeWX were incubated with both enzymes for 24 h. *Re*GH67 was not able to remove UA_me_ from the two UA_me_xylans (Fig. 1). In contrast, *Re*GH115 was able to release UA_me_ from both BiWX and BeWX. The release of UA_me_ from the xylan backbone led to aggregation. The latter was seen from the increase in higher molecular mass material (around 112.8 kDa) and from the decrease of lower molecular weight material (around 5.5 kDa) seen by HPSEC analysis of BiWX (Fig. 2). Such aggregation was expected, because the removal of UA_me_ led to larger blocks of unsubstituted xylosyl residues, which is known to allow self-association of these linear xylan blocks [19, 20].

As mentioned earlier in the text, in literature only four GH115 α-glucuronidases are described for their mode-of-action. All four were able to release UA_me_ from UA_me_xylan, as was observed in our study for *Re*GH115.

### Optimum temperature and pH

Activity of ReGH67 and ReGH115 towards 4-*O*-methylglucuronoxylan. ReGH67 showed a maximum release of UA_me_ from aldouronic acids (AAc) at a pH range from 4 to 6 and at a temperature range from 50 to 70 °C (Additional file 1: Figure S1). In case of ReGH115, the optimum release of UA_me_ from beechwood xylan (BeWX) was observed at pH 4 and at 65–70 °C. Considering these optima, for further hydrolysis experiments a pH of 4.0 at 70 °C was taken for both enzymes to allow best comparison.

### Mode-of-action of *Re*GH115 and *Re*GH67 towards AAc

**Fig. 3:**
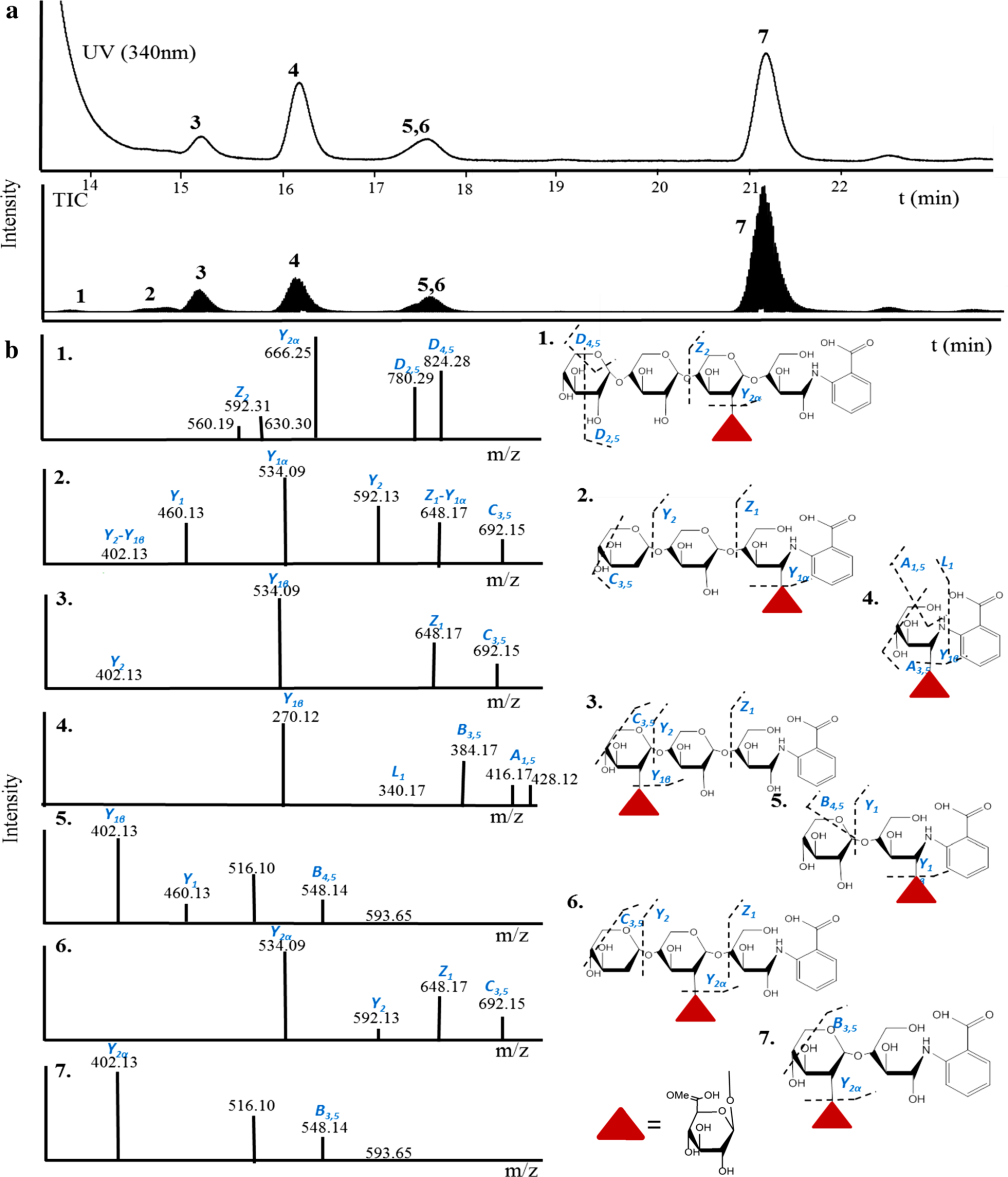
RP-UHPLC-UV-MS profiles [UV (340 nm) and total ion current (TIC)] of 2-AA-labelled AAc (**a**) and MS^2^ fragmentation spectra (**b**) of the in (**a**) annotated peaks: 1 XXU^4m2^ (856 m/z), 2,3,6 XXU^4m2^, U^4m2^XX, XU^4m2^X (724 m/z), 4 U^4m2^ (460 m/z), 5,7 XU^4m2^, U^4m2^X (592 m/z). *Filled triangle* 4-*O*-methylglucuronic acid

AAc is a commercially available mixture of various XOS with one UA_me_ linked per xylo-oligomer. The position of the UA_me_ in each oligomer is, however, not specified by the supplier. To enable the analysis of the mode-of-action of *Re*GH67 and *Re*GH115 towards characterised UA_me_XOS, first the exact structures of the UA_me_XOS present in AAc were determined. Hereto, AAc was labelled under reducing conditions with 2-AA and submitted to RP-UHPLC-MS analysis. The RP-UHPLC-UV absorbance at 254 and 340 nm showed the presence of six peaks (Fig. 3), while the number of peaks detected by total ion current (TIC) was eight. Seven out of the eight peaks detected by TIC were identified based on the recorded MS^2^ spectra, and the exact position of the UA_me_ for each of the XOS present in AAc was determined. The latter determination was performed by a separate analysis, in which the elution of the TIC analysed masses in 2-AA-labelled AAc was detected as single reaction monitoring (SRM). SRM was needed to overcome the problem of co-elution of certain structures, as shown in Fig. 3. Assisted by the separation, the detected parent mass, and the mass fragmentation pattern, seven structures were confirmed in AAc. These seven structures are depicted in Fig. 3, including their fragmentation patterns and masses. From Fig. 3, it is clear that UA_me_XOS are present having the UA_me_ linked to either the non-reducing, the internal, or the reducing end xylosyl residues. According to the nomenclature for UA_me_XOS structures [21], which uses ‘X’ for xylosyl residues and ‘U^4m2^’ for xylosyl residues attached via their *O*2 to a 4-*O*-methylglucuronic acid, the structures were named as 1 = XXU^4m2^X, 2 = XXU^4m2^, 3 = U^4m2^XX, 4 = U^4m2^, 5 = XU^4m2^, 6 = XU^4m2^X and 7 = U^4m2^X. The numbers correspond to the numbers shown next to the structures in Fig. 3.

**Fig. 4:**
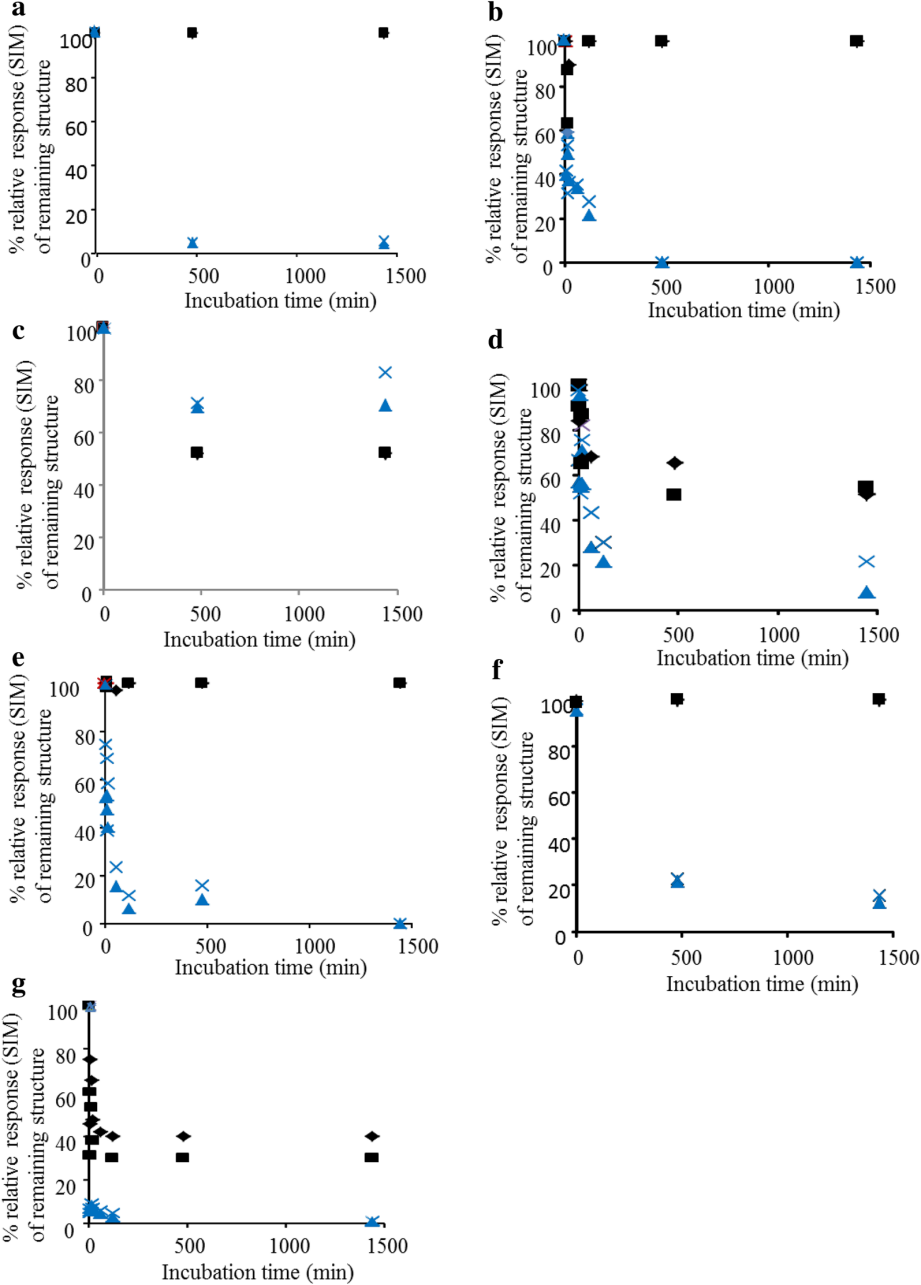
SRM relative abundance of AAc characterised structures during incubation with *Re*GH67 (*filled square* and *filled diamond*) or *Re*GH115 (x and *filled triangle*): 1 XXU^4m2^X (**a**), 2 XXU^4m2^ (**b**), 3 U^4m2^XX (**c**), 4 U^4m2^ (**d**), 5 XU^4m2^ (**e**), 6 XU^4m2^X (**f**) and 7 U^4m2^X (**g**). Experimental duplicates are displayed individually

Quantification of 2-AA-labelled UA_me_XOS in the *Re*GH67 and *Re*GH115 digests was achieved using single reaction monitoring (SRM; Fig. 4). It allowed the quantification of selected fragments as described in the “Methods” section. Figure 4 shows that *Re*GH67 only cleaved UA_me_ from the structures 3 (U^4m2^XX), 4 (U^4m2^) and 7 (U^4m2^X), which are substituted at the non-reducing end xylosyl residue. Hence, the mode-of-action of *Re*GH67 was comparable to those of many GH67 α-glucuronidases described previously [9–11, 22].

The activity of *Re*GH115 towards AAc is of particular interest, because its mode-of-action is expected to be a valuable contribution to the so far poorly described α-glucuronidases from the GH115 family. *Re*GH115 was able to release UA_me_ from *all* AAc structures, although U^4m2^XX mostly remained (Fig. 4). Therefore, it was concluded that *Re*GH115 did not show a distinct preference for a cleavage site in UA_me_XOS. UA_me_ is removed from xylose moieties at the reducing, internal, and non-reducing end side positions. So, under the conditions applied, clearly, *Re*GH115 degraded AAc to a further extent than *Re*GH67.

In terms of oligosaccharides, a summary of the mode-of-action of the four α-glucuronidases from family GH115 described in literature and of our *Re*GH115 is given in Table 1. In contrast to the absence of preference of *Re*GH115, the *S. commune* GH115 α-glucuronidase prefers to cleave UA_me_ from internal xylosyl residues of UA_me_XOS with a DP of 4–6 [16]. The *B. ovatus* GH115 α-glucuronidase prefers to hydrolyse UA_me_ from either internal or non-reducing end xylosyl residues in UA_me_XOS (DP 2–4) [13]. The *P. stipitis* GH115 α-glucuronidase cleaved the UA_me_ from UA_me_XOS in a DP range from 3 to 6 [23]. However, the preference for internal or outer units remains undefined in that research. The *S. pristinaespiralis* GH115 α-glucuronidase was only tested towards one UA_me_XOS having a DP of 5. To summarise, in comparison with the published activities of GH115 α-glucuronidases to date (Table 1), our *Re*GH115 showed activity towards UA_me_Xylan and UA_me_XOS and showed no preference for the position of the UA_me_ in the oligosaccharides tested.

**Table 1:** Performance of GH115 α-glucuronidases described in literature towards polymeric and oligomeric xylan substrates

α-glucuronidases	*Sc*Agu115	*Ps*Agu115	*Sp*Agu115	*Bo*Agu115	*Re*GH115
Organism	*Schizophyllum commune*	*Pichia stipitis*	*Streptomyces pristinaespiralis*	*Bacteroides ovatus*	*Rasamsonia emersonii*
Division	Basidiomycete	Ascomycete	Actinobacterium	Bacteroidete	Ascomycete
Mw (Kda)	125	120	nt	199	150
pH optimum	nt	4.4	nt	nt	4.0
T optimum (°C)	nt	60	nt	nt	65-70
3D-structure	No	No	No	Yes	No
Activity on xylan					
BeWX	nt	+	+	nt	+
BiWX	nt	nt	+	nt	+
SpW	+	nt	nt	nt	nt
WWX	nt	nt	nt	+	nt
Activity on U^4m2^X_n_^a^					
U^4m2^	nt	nt	nt	+	+
U^4m2^X	nt	nt	nt	+	+
XU^4m2^	+	+	nt	nt	+
U^4m2^XX	+	+	nt	+	+
XU^4m2^X	++	+	nt	++	+
XXU^4m2^	nt	nt	nt	nt	+
U^4m2^XXX	nt	nt	nt	++	nt
XU^4m2^XX	++	+	nt	nt	nt
XXU^4m2^X	nt	nt	+	nt	+
XXU^4m2^XX	++	+	nt	nt	nt

Active towards substrate (+), preferential degradation (++)

*nt* Not tested, *BeWX* Beechwood xylan, *BiWX* Birchwood xylan, *SpW* Spruce wood, *WWX* Willow wood xylan

*Sc*Agu115 Chong et al. [16]; Tenkanen and Siika-aho [15]; *Ps*Agu115 Ryabova et al. [14]; Kolenová et al. [23]; *Sp*Agu115 Fujimoto et al. [12]; *Bo*Agu115 Rogowski et al. [13]

^a^Nomenclature used is according to Faure et al. [21]

## Conclusion

Both *Re*GH67 and *Re*GH115 showed highest activity around pH 4 and at a temperature between 65 and 70 °C. *Re*GH67 released only UA_me_ attached to the xylosyl residue located at the non-reducing end of UA_me_XOS. *Re*GH115 was able to release UA_me_ from both UA_me_xylan and UA_me_XOS. It showed no preference for the position of the UA_me_ in the UA_me_XOS. This analysed mode-of-action is a valuable contribution to the so far poorly described α-glucuronidases from the GH115 family. Also, the knowledge presented is helpful to improve current enzyme cocktails for biorefinery applications.

## Methods

### Materials used

The aldouronic acid mixture (AAc) containing XOS of a DP of 2–5, having one UA_me_ substituent, was supplied by Megazyme (Wicklow, Ireland). Beechwood xylan (BeWX), BiWX and all chemicals used were purchased from Sigma-Aldrich (St Louis, MO, USA), unless otherwise specified. The carbohydrate composition of BeWX and BiWX was 68 and 69 % (w/w) xylan, respectively, and 9 % (w/w) UA_me_ for both substrates as determined elsewhere [18].

### Enzymes’ expression, production and purification

The two α-glucuronidases from *R. emersonii* (CBS 393.64), *Re*GH67 (LT555569) and *Re*GH115 (LT555570) [24], were expressed and produced in *A. niger* as described previously [25]. Purification of both *Re*GH67 and *Re*GH115 was carried out with a multiple-step chromatographic separation approach, as described in detail below, using an AKTA-explorer preparative chromatography system (GE Healthcare, Uppsala, Sweden). As a first step, 2 mL of the crude enzyme mixture (around 2 mg mL^−1^ protein) was subjected to a self-packed Superdex 200 26/60 column (GE Healthcare), pre-equilibrated in 20 mM Tris–HCl buffer (pH 7.0). After protein application, the column was eluted with three column volumes of buffer. Elution was performed at 6 mL min^−1^. The eluate was monitored at 214 and 280 nm. Fractions (4 mL) were collected and immediately stored on ice. Peak fractions were pooled and concentrated by ultrafiltration (Amicon Ultra, 10 kDa, Merck Millipore, Cork, Ireland) at 4 °C. The concentrated pools were subjected to SDS-PAGE and analysis of xylanase activity. Fractions close to the expected molecular mass (*Re*GH67 = 92 kDa; ReGH115 = 111 kDa) were re-submitted to Superdex 200 26/60 fractionation (2nd step). Now, the *Re*GH67-containing pool was devoid of xylanase activity. The *Re*GH115-containing pool was subjected to further purification (3rd step), and loaded onto a resource S column (30 × 16 mm i.d., GE Healthcare), pre-equilibrated with 20 mM sodium acetate buffer (pH 4.0). After protein application, the column was washed with 20 column volumes of starting buffer. Elution at 6 mL min^−1^ was performed with a linear gradient of 0–1 M NaCl in 20 mM sodium acetate buffer (pH 4) over 20 column volumes. Elution was monitored at 214 and 280 nm. Fractions (4 mL) were immediately stored on ice. Peak fractions were pooled, concentrated by ultrafiltration and subjected to SDS-PAGE. Fractions close to the expected molecular weight (111 kDa) were pooled to obtain *Re*GH115 and analysed for their protein content.

### Enzymatic hydrolysis

BeWX and BiWX were incubated with the purified α-glucuronidases (*Re*GH67 and *Re*GH115) in 10 mM NaOAc buffer, pH 4.0 (1 mL, 10 mg substrate dry matter) at 70 °C for 24 h. The enzymes were dosed at 0.05 % (w/w) protein per substrate added. To stop the enzyme incubations, 2 µl of 4 M HCl was added and the sample was centrifuged (10,000×*g*, 10 min, 10 °C) prior to analysis.

The pH and temperature optima were tested by incubating *Re*GH67 and *Re*GH115 with AAc and BeWX in a range of pH 2–7 using 200 mM NaOAc, and the temperatures ranging from 40 to 90 °C (Additional file 1: Figure S1). Incubation time, enzyme dose and stopping of the reaction was performed as described above.

The AAc was used as a substrate to determine the preferential substrate cleavage site of the enzymes over time. Hereto, 200 µL of sample was collected at various hydrolysis times: 0, 5, 10, 15, 20 and 60 min, 2, 8 and 24 h. All samples collected were submitted to HPAEC and, after 2-AA labelling, to RP-UHPLC-MS. The incubation was performed under the same conditions and protein and substrate concentrations as described above for the incubation with BeWX.

### Protein analysis

SDS-PAGE was performed using precast 8–16 % bis-acrylamide gradient gels (Bio-Rad, Hercules, CA, USA) in running buffer containing 25 mM Tris–HCl, 10.3 % (w/v) SDS at 100 V. The samples were denatured prior to loading to the gel at 95 °C for 5 min in a loading buffer containing 0.35 M Tris–HCl, 10.3 % (w/v) SDS, 36 % (v/v) glycerol, 5 % (v/v) 2-mercaptoethanol and 0.012 % (w/v) bromophenol blue (pH 6.8). The gels were stained using Coomassie brilliant blue. Protein content was determined according to Bradford [26].

### High-performance anion exchange chromatography (HPAEC)

Oligosaccharides and 4-*O*-methylglucuronic acids released after enzymatic incubation of AAc and BeWX with *Re*GH67 or *Re*GH115 were analysed by HPAEC as described elsewhere [1]. Quantification of 4-*O*-methylglucuronic acid was based on a calibration curve of glucuronic acid (0–50 µg mL^−1^).

### Reverse-phase ultra-high-performance liquid chromatography ultra violet–mass spectrometry (RP-UHPLC UV-MS)

A mixture of AAc, xylose, xylobiose, xylotriose and xylotetraose was labelled with anthranilic acid (2-AA) as described by Ruhaak [27] with some modifications: 50 µL of sample (2 mg mL^−1^) was dried under vacuum and mixed with 50 µL of a freshly prepared mixture (1:4) of 2-AA (192 mg mL^−1^) and 2-picoline borane (143 mg mL^−1^) in DMSO containing 30 % (v/v) of glacial acetic acid.

**Fig. 5:**
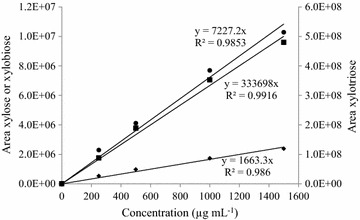
RP-UHPLC-UV (340 nm) response area of various concentrations of 2-AA-labelled xylose (*filled diamond*), xylobiose (*filled circle*) and xylotriose (*filled square*). The concentrations correspond to the amount subjected to labelling

AAc incubated with *Re*GH67 or *Re*GH115 were also labelled following the above-described procedure. All 2-AA-labelled samples were submitted to RP-UHPLC UV-MS analysis. Labelled oligomers were separated on a UHPLC Shield C18 BEH column (2.1 × 150 mm, 1.7 µm particle size; Waters, Milford MA, USA) using an Accela UHPLC system (Thermo Scientific, San Jose, CA, USA) equipped with a pump, degasser, autosampler and a photodiode array (PDA) detector, and coupled *in*-*line* to an LTQ-Velos double ion trap mass spectrometer equipped with a heated ESI probe (Thermo Scientific). The eluents were 0.1 % (v/v) formic acid in demineralised water (A), 0.1 % (v/v) formic acid in acetonitrile (B) and 50 % (v/v) acetonitrile in demineralised water (C). The flow rate was 300 µl min^−1^ and the sample injection volume was 10 µl. The elution programme was started at 95 % (v/v) A, 8 % (v/v) B for 25 min, followed by 25–35 min linear gradient to 80 % (v/v) A, 20 % (v/v) B; 35–36 min linear gradient to 50 % (v/v) A, 50 % (v/v) B; 36–41 min 50 % (v/v) A, 50 % (v/v) B; 41–42 min linear gradient to 92 % (v/v) A, 8 % (v/v) B; 42–50 min 92 % (v/v) A, 8 % (v/v) B. Re-equilibration was performed for 17 min at starting condition. The eluate was measured at 254 nm [28]. The compounds eluted were detected by MS in negative mode. Source heater temperature was set at 225 °C and the capillary temperature was 350 °C. Ion source voltage was set at −4.5 kV. The detected mass range was 300–2000 Da. MS^2^ was performed on the most intense ion detected, with normalised collision energy of 30 (arbitrary units). Single reaction monitoring (SRM) was acquired using the above-mentioned MS settings. The main MS^2^ fragments of each individual compound present in AAc were monitored together with the parent mass and retention time of the compound and given in Table 2. The most abundant fragments from each RP18-separated single mass were determined (Table 2). In a separate analysis, these most abundant fragments, which are considered to be the fingerprint of the structure, were selectively monitored. From the latter analysis, the sum of the areas of the two main fragments was used for quantification of the structures. In addition, increasing concentrations of 2-AA-labelled standards (xylose, xylobiose and xylotriose) were analysed. Samples were assumed to be labelled equally efficient as the standards, which showed a linear correlation (R^2^ = 0.99) of their UV response area (340 nm) upon increasing concentrations, as shown in Fig. 5.

**Table 2:** Single reaction monitoring (SRM) settings of AAc analysed by UHPLC-MS

Segment 1 (0–16 min)					Segment 2 (16–50 min)				
Scan event name	Parent mass (*m*/*z*)	Main MS^2^ fragment (F)		Rt (min)	Scan event name	Parent mass (*m*/*z*)	Main MS^2^ fragment (F)		Rt (min)
		F1 (*m*/*z*)	F2 (*m*/*z*)				F1 (*m*/*z*)	F2 (*m*/*z*)	
TIC	50–1000	na	na	na	TIC	50–1000	na	na	na
SRM 1	856	666	780	14	SRM 1	460	270	384	16.2
SRM 2	724	533	648	14.7	SRM 2	724	534	648	17.5
SRM 2	724	533	648	15.3	SRM 3	592	402	516	17.5
SRM 3	na	na	na	na	SRM 3	592	402	516	21.2

*Rt* retention time in minutes,* na* not applicable

## Additional file

10.1186/s13068-016-0519-9 pH (A) and temperature profiles (C) of the incubated *Re*GH67 with the aldouronic acids mixture (AAc) and the pH (B) and temperature profiles (D) of the incubated *Re*GH115 with beechwood xylan.

## Abbreviations

2-AA: anthranilic acid
AAc: aldouronic acids
BeWX: BeechWood glucuronoXylan
BiWX: BirchWood glucuronoXylan
CAZy: Carbohydrate-Active enZymes
GH: glycoside hydrolases
HPAEC: high-performance anion exchange chromatography
MALDI-TOF MS: matrix-assisted laser desorption/ionisation time-of-flight mass spectrometry
PAD: pulsed amperometric detection
PDA: photodiode array
SDS-PAGE: sodium dodecyl sulphate polyacrylamide gel electrophoresis
SRM: single reaction monitoring
UA_me_: 4-*O*-methyl-glucuronic acid
UA_me_XOS: 4-*O*-methyl-glucuronic acid-substituted xylo-oligosaccharides
*Re*: *Rasamsonia emersonii*
RP-UHPLC UV-MS: reverse-phase ultra-high-performance liquid chromatography–ultraviolet mass spectrometry
XOS: xylo-oligosaccharides
