# Supplementary figures and images for: The two Rasamsonia emersonii α-glucuronidases, ReGH67 and ReGH115, show a different mode-of-action towards glucuronoxylan and glucuronoxylo-oligosaccharides
Source: Biotechnol Biofuels. 2016 May 18;9:105. doi: 10.1186/s13068-016-0519-9 (PMC4870768; doi:10.1186/s13068-016-0519-9)

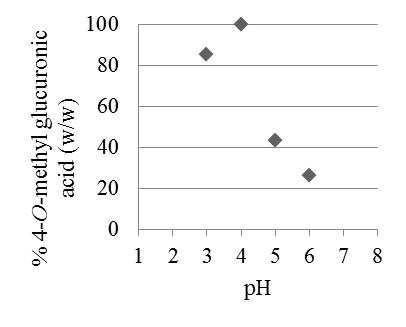

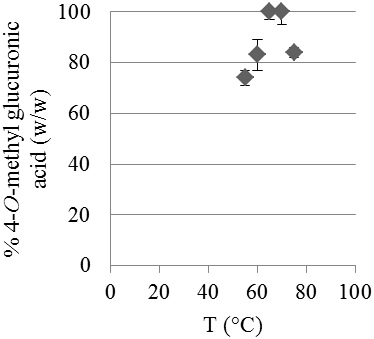

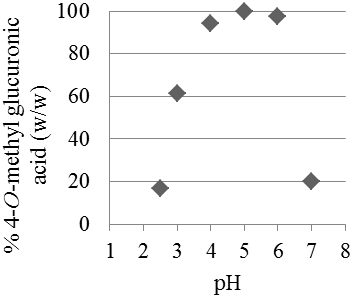

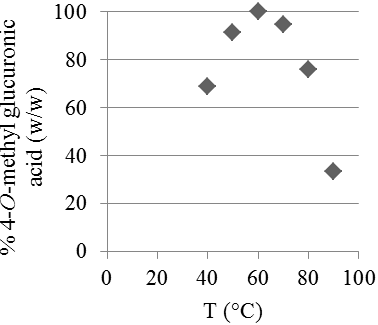

Supplement: Supplementary file 1 — 10.1186/s13068-016-0519-9 pH (A) and temperature profiles (C) of the incubated ReGH67 with the aldouronic acids mixture (AAc) and the pH (B) and temperature profiles (D) of the incubated ReGH115 with beechwood xylan. [file 13068_2016_519_MOESM1_ESM.docx]
